# Clock Walking and Gender: How Circular Movements Influence Arithmetic Calculations

**DOI:** 10.3389/fpsyg.2018.01599

**Published:** 2018-09-25

**Authors:** Luisa Lugli, Stefania D’Ascenzo, Anna M. Borghi, Roberto Nicoletti

**Affiliations:** ^1^Department of Philosophy and Communication, University of Bologna, Bologna, Italy; ^2^Department of Dynamic and Clinical Psychology, Sapienza University of Rome, Rome, Italy; ^3^Institute of Cognitive Sciences and Technologies, Italian National Research Council, Rome, Italy

**Keywords:** embodied cognition, body motion, arithmetical calculations, circular motion, gender differences

## Abstract

Starting from a rich body of evidence on the strict bidirectional relationship between numerical cognition and action processes, the present study aims at deepening the existing knowledge of the influence of body movement on arithmetic calculation. Numerous studies have shown that moving the body along the vertical or the horizontal axis could facilitate calculations such as additions and subtractions. More specifically, results showed an effect of congruence between the type of operation (additions vs. subtractions) and the direction of the movement performed (up/right or down/left). While this congruence effect is present for both additions and subtractions when the axis of action is vertical, when the axis of action is horizontal, the effect appears only for additions. The purpose of this study is to investigate the influence of circular motion, which has so far not been explored, on counting. Participants were asked to count by adding or subtracting “three,” while performing a circular motion (i.e., a clockwise or counterclockwise movement), in an active (i.e., walking) or passive mode (i.e., being pushed on a wheelchair). Results showed a congruence effect for additions calculated in the active modality and only for male participants. Implications of the results for theories of embodied cognition and for the debate on gender differences in mathematical skills are discussed in this paper.

## Introduction

In the last two decades, a growing number of studies have increased our knowledge about the link between space and numerical cognition. The seminal effect demonstrating an influence of number magnitude on space is the so-called spatial–numerical association of response codes (SNARC) effect (Dehaene et al., 1993). In a typical task leading to this effect, participants are required to make an odd–even classification of numbers from 1 to 9 by pressing a left or right key. Typical results show faster and more accurate left responses following small numbers (e.g., two) and right responses following large numbers (e.g., nine) as compared with opposite instruction. This evidence led Dehaene and colleagues to suggest the existence of a horizontal mental number line. Accordingly, numbers would be represented, at least for Western cultures, along a continuum from left to right, according to their magnitude (see Zorzi et al., 2002; Hubbard et al., 2005; Wood et al., 2008; see also for another account Proctor and Cho, 2006).

Two main research lines are relevant to the present paper: the first highlights the relevance of space, the second of the motor system for numerical cognition. In the first research line, several studies, starting from the finding of Dehaene et al. (1993) of a spatial–numerical association for elementary number processing, investigated whether the same spatial biases emerge also during arithmetic calculation. Different paradigms were used in order to demonstrate a link between space and arithmetic calculations. For example, Pinhas and Fischer (2008) asked participants to point where the digit, resulting from arithmetic problem, was located on a number line, while Knops et al. (2009) observed where participants pointed the mouse to indicate the more plausible results of an arithmetic problem choosing among seven different positions on the screen. In both studies, a bias to the right or to the left was observed when the problem was an addition or a subtraction, respectively. The SNARC effect has been interpreted differently, either as a phenomenon concerning attention (e.g., Dodd et al., 2008), or as a phenomenon engaging both attention and body action (e.g., Shaki and Fischer, 2014). We will focus on this second interpretation that we will discuss while presenting the second research line.

The second research line starts from an embodied and grounded view, i.e., from the view according to which cognition is grounded in sensorimotor systems (e.g., Barsalou, 2008, 2016; Glenberg, 2015; Borghi and Caruana, 2016). Several studies adopting this perspective have been dedicated to investigating whether numerical cognition might also be affected by the activation of the motor system. This influence is explained by the fact that the spatial–numerical association would emerge from learning to count using our fingers (Fischer and Brugger, 2011). It has been demonstrated that the relation between numerical cognition and action-related processes is bidirectional. Several studies have observed the influence of processing arithmetic calculation on action-related processes (e.g., Wiemers et al., 2014; Hartmann et al., 2015, 2016); conversely, a number of studies report influence of body part movements on arithmetic calculation (e.g., Lugli et al., 2013; Anelli et al., 2014; Masson and Pesenti, 2014). Importantly, also the mode through which the movement is experienced (passive mode) or performed (active mode) could influence the relation between numerical cognition and action-related processes (Hartmann et al., 2012a,b; Lugli et al., 2013). For example, Hartmann et al. (2012b) asked participants to generate numbers at random, while they were seated in a chair positioned on a platform moving along the transversal, frontal, and sagittal body planes. They found a numerical bias for small numbers when the passive motion experienced by the participants was leftward or downward (Experiment 1). Furthermore, an influence of the passive motor direction on number processing emerged also when the number magnitude information is not relevant for the task (Hartmann et al., 2012a). To the best of our knowledge, only one study so far, has compared the passive and active motions during arithmetic calculations. More specifically, recent findings of our group have shown that bodily movements along the vertical axis (Lugli et al., 2013) influenced the correct performing of additions and subtractions only when the ascending/descending body motions were passive (i.e., taking the elevator). Participants indeed performed a greater number of operations when the spatial orientation associated with the type of calculation (e.g., for the additions: orientation up or right; for subtractions: orientation down or left; Lakoff and Núñez, 2000; Fischer, 2012) was congruent with the direction of the movement experienced with the body. These results suggest that participants experiencing and/or performing the motion can differently influence the numerical cognition and actions-related process relation, at least for the vertical and the horizontal axes.

Interestingly, this congruency effect emerged for both additions and subtractions only when the axis of action was vertical (Lugli et al., 2013), while when the axis of action was horizontal, the effect only appeared for additions (Anelli et al., 2014). These findings were in line with the claim of Wiemers et al. (2014) that congruence between movements and arithmetic calculations is more reliable for the vertical axis than the horizontal one: “mental calculations operate on representations on numerical magnitude that are grounded in a vertically organized mental number space” (ibidem, p.1). Recently, it has been claimed that while the horizontal axis is more influenced by cultural factors (e.g., the direction of writing) and is likely an artifact of measurement, number concepts are instead clearly associated to the vertical axis, in line with an embodied cognition perspective (Shaki and Fischer, 2018).

Starting from these results, the aim of the present study was to deepen the relationship between the passive or active movements of our body in space and the numerical processing by studying a movement that has so far not been explored, that is, the circular motion (i.e., clockwise/counterclockwise movement). The circular motion is a kind of axis we are very familiar with: everyday, for example, we experience the clockwise movements of time. Despite the prominent role of the vertical axes, evidence has shown that our experience of the clock-face circular motion can be very influential. Bächtold et al. (1998) demonstrated that just asking participants to conceive the centrally presented numbers as hours on a clock face is sufficient to reverse the so-called SNARC effect. However, one could argue that we do not experience circular movements as often as we experience horizontal movements or vertical ones: we usually do not walk continuously in a clockwise or counterclockwise direction, but we often turn toward the right or left, and go upstairs or downstairs from stairs, buses, elevators etc. Surprisingly, recent findings demonstrated that this type of movement also influences our perception. For example, Topolinski and Sparenberg (2011) demonstrated that moving a cranks in a clockwise direction induces psychological states of temporal progression and, accordingly, motivational orientations toward the future and novelty. Based on these findings, we can hypothesize that the clockwise movement direction maps onto the future, and probably also that counterclockwise movement direction maps onto the past. Other studies have demonstrated that thinking about the future has been associated with moving forward, and thinking about the past with moving backward (Miles et al., 2010a,b; Sell and Kaschak, 2011), even if the effect might vary depending on the culture (Núñez and Sweetser, 2006).

Regarding the relationship between spatial cognition and numerical processing, distance-based effects were found (for a review see Winter et al., 2015). For example, it has been demonstrated that responses to small numbers are faster with a nearer response button, while responses to large numbers are faster with a farther response button (e.g., Holmes and Lourenco, 2012; Shaki and Fischer, 2012). More interestingly for our purpose, to the best of our knowledge, no strong empirical evidence so far has related number magnitude and moving backward–forward in space. Indeed, Hartmann et al. (2012b) hypothesized that moving forward could be associated with higher numbers, whereas moving backward could be associated with smaller numbers. Despite these hypotheses, they did not find strong results, but a tendency to freely generate small numbers, while perceiving a backward motion and large numbers when perceiving a forward motion.

Based on the reviewed literature, we can formulate the following hypotheses. We expect a facilitation for additions with clockwise movement (thinking about the future, moving forward), and a facilitation for subtractions with counterclockwise movement (thinking about the past, moving backward). Additions namely imply an increase of quantity and, therefore, lead to larger numbers, while subtractions imply a decrease of quantity and thus yield small numbers.

Finally, we are interested in exploring if a difference may emerge depending on the gender of the participant performing the task. Gender differences have been observed in various types of activity: for example, the effects of gaze-cueing are more evident in females than in males (e.g., Bayliss et al., 2005) and females are more sensitive to social stimuli with respect to males (e.g., Lugli et al., 2017; Geary, 2010 for a review). However, the presence, the degree, and the origin of a gender difference in mathematical skills are still debated (e.g., Benbow and Stanley, 1980; Guiso et al., 2008; Hyde et al., 2008; Rumiati, 2010). This issue has been addressed on the one hand by hypothesizing that the difference between men and women has a biological basis (e.g., Kimura, 1999; Baron-Cohen, 2003; Roberts et al., 2008; Hatta and Nagaya, 2009; Colzato et al., 2010; Pletzer et al., 2014), and, on the other hand, by analyzing the stereotype according to which males would be better in mathematical tasks and would consider mathematical skills more important than women (e.g., Furnham et al., 2002; Li, 2004), and females would tend to perform badly in the presence of males (Inzlicht and Ben-Zeev, 2000). For the purpose of this study, it is particularly important to note that the analysis of the influence of individual differences on the link between spatial and numerical cognition has mainly focused so far on age (e.g., Wood et al., 2008; Hoffmann et al., 2014), on the mathematical expertise (Cipora et al., 2016), and on the abilities in mental rotation tasks (Viarouge et al., 2014), considering to a lesser extent of how this association may be different between men and women. A very recent paper has focused on the individual differences influencing symbolic magnitude comparison, showing that females reported slower reaction times and a larger unit–decade compatibility effect than male participants (Huber et al., 2017); importantly, the result was obtained in an online study, where the identity of participants can be hidden. Interestingly, Bull and colleagues (Bull and Benson, 2006; Bull et al., 2013) conducted a study to observe if gender could be a source of differences in numerical–spatial associations. In four experiments, authors found a difference between male and female participants by means of the SNARC effect, the numerical distance effect, and the number-line estimations. More specifically, Bull et al. (2013) consistently found that association between numerical and spatial representations is more evident in male than in female participants. However, these results do not imply a general better number-processing capability in men as compared with women. They leave the question open as to whether individuals of different gender may use different strategies to complete complex mathematical tasks and whether body movement differently influences men and women, due also to the documented gender difference in spatial cognition (Geary et al., 2000). These findings suggest a gender difference for number representation in adults; however, it is less known whether such a difference should emerge when participants are required to process not only the absolute numerical information (e.g., three rather than nine), but also to perform arithmetic calculations during whole body motions. Despite the growing number of studies focusing on arithmetic calculation during bodily actions, there is little in the extant literature that addresses this issue in adult participants as regarding gender differences. The present study is specifically designed to investigate gender differences in the effect of circular movement on arithmetic performance.

## Materials and Methods

### Participants

Forty-six students^1^ of the University of Bologna (23 females, *M_age_* = 21.35, *SD* = 1.23) took part in the experiment. The majority of participants had a background in humanities, and they were all naïve as to the purpose of the experiment.

### Ethics Statement

The experiment was approved by the Psychology Department’s ethical committee of the University of Bologna, and participants provided a written informed consent.

### Apparatus and Stimuli

Participants were asked to keep adding or subtracting three to a starting number (e.g., 371) for 22 s and say the result of each calculation aloud (e.g., 374, 377, 380 or 368, 365, 362, and so on, for additions and subtractions, respectively, until the 22 s elapsed). We made sure that the starting numbers: (a) were always composed by three digits (e.g., 371 and 587) and (b) started with two different digits (i.e., 3 or 5, such as 371 or 588).

### Procedure

As in Lugli et al. (2013) and Anelli et al. (2014), participants were required to make the calculations (additions or subtractions) while they were performing a movement, specifically in this case, while they were performing a circular movement inside a gym.

The experiment consisted of two blocks, one in which participants were sitting on a wheelchair (passive mode) and one where participants were walking along with the experimenter (active mode). Within each block, participants were required to perform four trials, resulting from the combination of the two types of calculation (i.e., additions and subtractions) and the two types of motion (i.e., clockwise and counterclockwise). We designed each block in order to make additions and subtractions always alternate (i.e., an addition always followed a subtraction and vice versa).

At the beginning of the motion, the experimenter spoke aloud the starting number and a go signal followed. Immediately after the go signal, the participant had to repeat the starting number and then had to say aloud the result of each calculation for 22 s consecutively until a stop signal was given. Therefore, the number of calculations made within the 22-s window entirely depended on the participants’ calculation speed. If the participant made a calculation error, the trial was stopped and a new trial started over and the participant had to choose a different starting number. No feedback of any kind was given during the calculations. Instructions stressed the importance of accuracy over speed.

**FIGURE 1:**
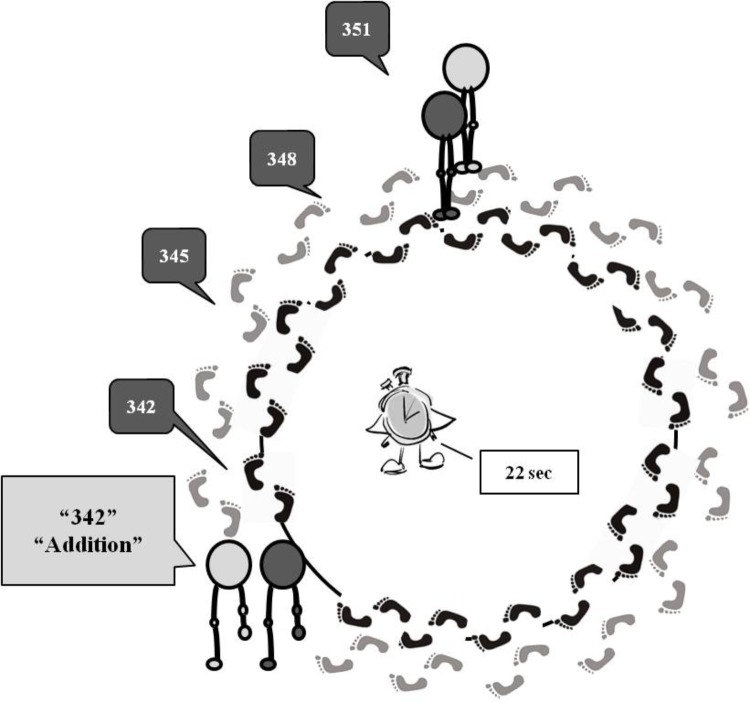
Schematic representation of the experimental procedure. At the beginning of each trial, the experimenter and the participant walked close to each other along a circular path. Then, the experimenter spoke aloud the starting number, the type of calculation to be executed, the direction of the movement to perform, and then she gave the go signal. Immediately after the go signal, participants started to walk, for example, clockwise, repeating the starting number and then saying the result of each calculation aloud, until the stop signal.

A circular path (diameter: 3.5 m) was drawn on the floor of the gym in order to easily signal the exact circular path all participants have to follow. In the passive mode condition, the experimenter pushed a wheelchair along the circular path where the participants were required to settle in at the beginning of the experiment. In the active mode condition, the experimenter and the participants walked side by side while performing the circular path (see **Figure 1**). The position of the experimenter was counterbalanced between participants.

Responses were recorded by the experimenter, who kept track and note of the starting number assigned to the participants and of the final number reached at the end of the 22-s time window.

Participants were thanked and debriefed at the end of the experiment.

## Results^2^

We considered the number of correct calculations as our dependent variable. When an error was made, that is, when participants made a wrong addition or subtraction, a penalty was considered: subtracting 0.5 to the amount of the total correct calculation made.

The number of calculations was entered into a repeated-measures ANOVA with type of operations (addition vs. subtraction), direction of the movement (clockwise vs. counterclockwise), and mode (active vs. passive) as within-subject factors and the gender (female vs. male) as between-subject factor. The magnitude of size effect was expressed by $\eta_{p}^{2}$. When necessary, *post hoc* comparisons were performed using paired samples *t*-tests and by correcting the *p*-value on the basis of the number of planned comparisons (Bonferroni correction).

The main effect of type of operation was significant [*F*(1,44) = 70.37, MSE = 2.060, *p* < 0.001, $\eta_{p}^{2}$ = 0.615]. The number of calculations was higher when participants performed additions (*M* = 10.5, *SD* = 2.9) with respect to subtractions (*M* = 9.3, *SD* = 2.9). The main effect of mode was significant [*F*(1,44) = 9.216, MSE = 2.031, *p* = 0.004, $\eta_{p}^{2}$ = 0.173]. The number of calculations was higher when participants performed calculations during the passive mode (i.e., wheelchair, *M* = 10.1, *SD* = 3.1) with respect to active mode (i.e., walking, *M* = 9.7, *SD* = 2.7). The main effect of gender was significant [*F*(1,44) = 15.65, MSE = 50.247, *p* < 0.001, $\eta_{p}^{2}$ = 0.262]. Male participants made more calculations (*M* = 11.4, *SD* = 2.9) compared to female ones (*M* = 8.4, *SD* = 1.9).

**FIGURE 2:**
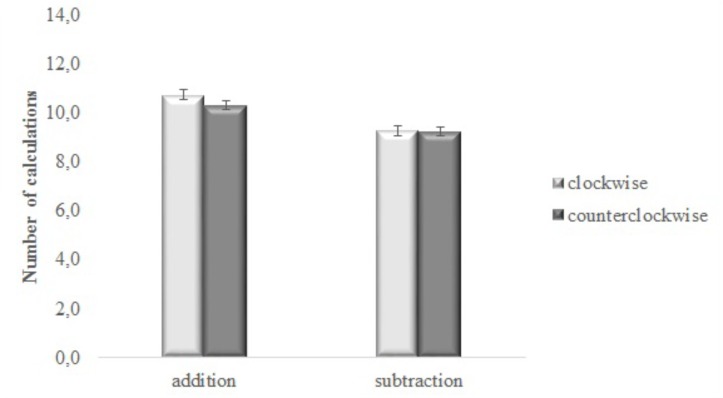
Number of calculations for addition and subtraction performed moving in the clockwise and the counterclockwise direction. Bars are standard error of the means (SEMs).

The interaction between type of operation and direction of the movement resulted as significant [*F*(1,44) = 4.347, MSE = 0.903, *p* = 0.043, $\eta_{p}^{2}$ = 0.090]. Paired-samples *t*-tests showed that the number of additions was higher when participants performed the clockwise movement (*M* = 10.8, *SD* = 3.2) as compared with the counterclockwise one (*M* = 10.3, *SD* = 2.8) [*t* (45) = 2.610, *p* = 0.012 (Bonferroni-corrected *p* level = 0.025)], while the number of subtractions was the same for the two movement’s directions (*M* = 9.3, *SD* = 3.1, *M* = 9.3, *SD* = 2.8 for clockwise and counterclockwise movements, respectively) [*t* (45) = 0.109, *p* = 0.914]; see **Figure 2**.

Furthermore, the interaction between the mode and the gender factor was significant [*F*(1,44) = 4.816, MSE = 2.031, *p* = 0.034, $\eta_{p}^{2}$ = 0.099]. Independent-samples *t*-tests showed, confirming the significant main effect of gender, that male participants performed more calculations than female participants for both the active and the passive movements (male: *M* = 11, *SD* = 2.7 and *M* = 11.8, *SD* = 3.3; female: *M* = 8.4, *SD* = 2.1 and *M* = 8.5, *SD* = 1.9; for the active and passive movements, respectively) [*t*_s_ (44) > 3.62, *p*_s_ < 0.001] (Bonferroni-corrected *p* level = 0.0125). Furthermore, paired sample *t*-test showed that while male participants performed more calculations in the passive mode compared with the active one [*t* (22) = 3.25, *p* = 0.004] (Bonferroni-corrected *p* level = 0.0125), for female participants, no significant difference emerged [*t* (22) = 0.706, *p* = 0.487].

Neither other main effect nor other interactions resulted as significant [*F*_s_ < 3.14, *p*_s_ > 0.083, *n*_p_^2^_s_ < 0.067].

**FIGURE 3:**
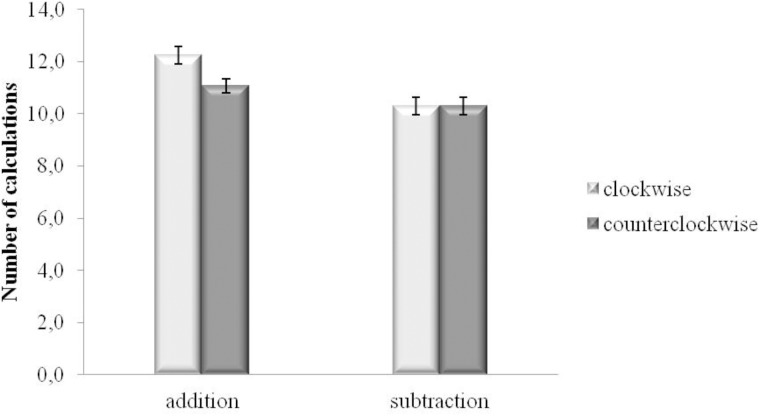
Number of calculations for addition and subtraction performed walking in the clockwise and the counterclockwise directions for the male participants. Bars are standard error of the means (SEMs).

Separate analyses by levels of gender (for a support on the split-sample analysis by gender, see Lange-Küttner, 2017) showed that the type of operation X direction of the movement was significant for male participants [*F*(1,22) = 5.306, MSE = 0.832, *p* = 0.031, $\eta_{p}^{2}$ = 0.194], but not for the female ones [*F*(1,22) = 0.504, MSE = 0.973, *p* = 0.485, $\eta_{p}^{2}$ = 0.022]. More specifically, as regard male participants, the interaction was nearly significant in the active mode modality condition [*F*(1,22) = 4, MSE = 1.767, *p* = 0.058, $\eta_{p}^{2}$ = 0.154], and not significant in the passive mode condition [*F*(1,22) = 0.084, MSE = 1.166, *p* = 0.775, $\eta_{p}^{2}$ = 0.004]. Paired-samples *t*-tests, replicating the results of the main ANOVA, showed that male participants calculated more additions when they were walking in the clockwise direction (*M* = 12.2, *SD* = 3.2) as compared with the counterclockwise direction (*M* = 11.1, *SD* = 2.6) [*t* (22) = 3.219, *p* = 0.004] (Bonferroni-corrected *p* level = 0.0125), while the number of subtractions was the same in both directions (*M* = 10.3, *SD* = 3.2 and *M* = 10.3, *SD* = 3.2, for clockwise and counterclockwise movements, respectively) [*t* (22) = 0.141, *p* = 0.889]. Furthermore, results showed that when male participants performed the clockwise movement, additions were facilitated with respect to subtractions (*M* = 12.2, *SD* = 3.2 and *M* = 10.3, *SD* = 3.2, for additions and subtractions, respectively) [*t* (22) = 4.123, *p* < 0.001] (Bonferroni-corrected *p* level = 0.0125). The same was not true for the counterclockwise movement direction: paired-samples *t*-tests did not show significant difference between the number of additions (*M* = 11.1, *SD* = 2.6) and subtractions (*M* = 10.3, *SD* = 3.2) [*t* (22) = 1.798, *p* = 0.086], see **Figure 3**.

## Discussion

Results showed the presence of a relationship between the type of arithmetic calculation (addition and subtraction) and the direction of the circular movement performed (clockwise and counterclockwise). This result emerged regardless of whether the participant was moving in space in an active (walking) or passive mode (being pushed on a wheelchair). However, the effect only emerged for additions and not for subtractions, replicating, with the circular movements, the findings of Anelli et al. (2014) on the horizontal axis. The choice to investigate the circular motion came from the assumption that moving in a clockwise or counterclockwise direction could be perceived as a strengthened horizontal movement. Let us imagine that performing a clockwise motion could be perceived as to continue to turn rightward, while performing a counterclockwise motion could be perceived as to continue to turn leftward. Following this rationale, the clockwise and counterclockwise circular movements might represent a kind of empowerment of horizontal movements to the right and to the left, respectively. For this reason, we expected a facilitation effect also for the subtractions; however, this was not to be the case. Probably the reason why moving horizontally (Anelli et al., 2014) or circularly in space did not affect the calculation of subtractions depends on the presence of different mechanisms subtending additions and subtractions (e.g., Domahs and Delazer, 2005; Barrouillet et al., 2008).

The novelty of the present study concerns two main points.

First, this is the first study demonstrating the relationship between arithmetic calculation and circular movements in space. We found facilitation for additions, which imply an increase of quantity and, therefore, lead to larger numbers, with clockwise movement (thinking about the future, moving forward), suggesting that additions map onto the future and moving forward. These findings could give initial support to the idea of the existence of a link between time lines and arithmetic calculations. In the same vein, Matlock et al. (2011) showed that counting upward/downward influences the reasoning about time. However, whether an interaction between the spatial mapping of time and numerical cognition exists requires further investigations.

Second, as far as we know, this is the first study that explores the relationship between space and arithmetic calculations, taking into consideration gender differences. Results showed a main effect of the gender factor pointing out that male participants perform overall more arithmetic calculations as compared with female participants. This is supported also by the significant interaction between gender and mode factors, demonstrating that male participants performed more calculations than female ones in both active and passive movements. More interestingly, it seems that the effect due to the direction of movement (clockwise and counterclockwise) and the type of arithmetic calculation (addition and subtraction) is present only in male participants. These results add new empirical evidence to the debate on socio-cultural stereotypes according to which males would be better than females in both mathematical and spatial abilities (e.g., Halpern et al., 2007; for different results see Huber et al., 2017). Furthermore, these results show that the best performance emerges when male participants performed these two activities at the same time.

To conclude, our findings suggest that the arithmetic abilities we implement in several contexts of everyday life (e.g., when calculating how far is the next bus station) are in close relationship with the movements we perform with our body. The importance of bodily experiences for spatial–numerical associations and acquisition of mathematical knowledge has been recently underlined also in the educational field. Recent empirical studies have shown that using gestures facilitates math-knowledge acquisition (e.g., Goldin-Meadow et al., 2001, 2009). Furthermore, several studies used whole body spatial movement to train spatial–numerical associations in children (e.g., Fischer et al., 2011, 2015; Link et al., 2013), significantly improving spatial–numerical mappings and increasing arithmetic skills in children. Further research is needed to explore the relationship between movement in space and both learning and development of counting abilities.

## Author Contributions

Testing and data collection were performed by SD and LL. SD and LL performed the data analysis and interpretation under the supervision of AB and RN. LL wrote the main manuscript text. All authors developed the study concept, contributed to the study design, reviewed the manuscript, and approved the final version of the manuscript for submission.

## Conflict of Interest Statement

The authors declare that the research was conducted in the absence of any commercial or financial relationships that could be construed as a potential conflict of interest.

## Footnotes

1 We calculated the sample size required to achieve 95% power to detect a significant 2 Type of operations (addition vs. subtraction), ×2 Direction of the movement (clockwise vs. counterclockwise) ×2 Mode (active vs. passive) ×2 Gender (female vs. male) interaction effect using G^∗^power 3.1 (Faul et al., 2007) software. Using an effect size *f* = 0.6 (Cohen, 1988), the power calculation yielded a recommended sample size of 34 participants.

2 These data have been already partially published as a brief report in the Italian journal *Giornale Italiano di Psicologia*.
